# Physiologically Based Pharmacokinetic Modelling to Predict Pharmacokinetics of Enavogliflozin, a Sodium-Dependent Glucose Transporter 2 Inhibitor, in Humans

**DOI:** 10.3390/pharmaceutics15030942

**Published:** 2023-03-14

**Authors:** Min-Soo Kim, Yoo-Kyung Song, Ji-Soo Choi, Hye Young Ji, Eunsuk Yang, Joon Seok Park, Hyung Sik Kim, Min-Joo Kim, In-Kyung Cho, Suk-Jae Chung, Yoon-Jee Chae, Kyeong-Ryoon Lee

**Affiliations:** 1 College of Pharmacy, Seoul National University, Seoul 08826, Republic of Korea; 2 Laboratory Animal Resource Center, Korea Research Institute of Bioscience and Biotechnology, Cheongju 28116, Republic of Korea; 3 Life Science Institute, Daewoong Pharmaceutical, Yongin 17028, Republic of Korea; 4 School of Pharmacy, Sungkyunkwan University, Suwon 16419, Republic of Korea; 5 Research Institute of Pharmaceutical Sciences, Seoul National University, Seoul 08826, Republic of Korea; 6 College of Pharmacy, Woosuk University, Wanju-gun 55338, Republic of Korea; 7 Department of Bioscience, University of Science and Technology, Daejeon 34113, Republic of Korea

**Keywords:** enavogliflozin, DWP16001, GCC5694A, sodium-glucose cotransporter 2 inhibitor, diabetes mellitus, physiologically based pharmacokinetic modelling, pharmacokinetics, mechanistic kidney model, in vitro–in vivo extrapolation

## Abstract

Enavogliflozin is a sodium-dependent glucose cotransporter 2 (SGLT2) inhibitor approved for clinical use in South Korea. As SGLT2 inhibitors are a treatment option for patients with diabetes, enavogliflozin is expected to be prescribed in various populations. Physiologically based pharmacokinetic (PBPK) modelling can rationally predict the concentration–time profiles under altered physiological conditions. In previous studies, one of the metabolites (M1) appeared to have a metabolic ratio between 0.20 and 0.25. In this study, PBPK models for enavogliflozin and M1 were developed using published clinical trial data. The PBPK model for enavogliflozin incorporated a non-linear urinary excretion in a mechanistically arranged kidney model and a non-linear formation of M1 in the liver. The PBPK model was evaluated, and the simulated pharmacokinetic characteristics were in a two-fold range from those of the observations. The pharmacokinetic parameters of enavogliflozin were predicted using the PBPK model under pathophysiological conditions. PBPK models for enavogliflozin and M1 were developed and validated, and they seemed useful for logical prediction.

## 1. Introduction

Glucose is a critical substrate of metabolism in eukaryotic organisms, and the homeostasis of blood glucose levels is essential for preventing metabolic disorders, including diabetes. Glucose is freely filtered through the renal glomerulus and enters the tubular system of the kidney. However, in healthy individuals, filtered glucose is almost completely reabsorbed in the proximal tubule. Therefore, glucose is absent or present at very low concentrations in the urine, and the loss of glucose is minimized.

Sodium-dependent glucose cotransporters (SGLTs) mediate glucose reabsorption against concentration gradients by coupling glucose transport and sodium transport. There are two well-known types of SGLTs in the kidney: SGLT1 and SGLT2 [1]. SGLT1 plays a minor role in renal glucose reabsorption [2]. Simultaneously, most of the glucose reabsorption in the kidney is mediated by the SGLT2, primarily localized in the S1 segment of the proximal tubule in the kidney [1,2,3,4]. Therefore, SGLT2 inhibitors have recently emerged as one of the most promising glucose-lowering therapeutic agents. By selectively inhibiting the tubular reabsorption of glucose, SGLT2 inhibitors promote the urinary excretion of glucose and lower blood glucose levels [1].

Enavogliflozin (DWP16001) is a selective SGLT2 inhibitor developed by Daewoong Pharmaceutical Co., Ltd. (Seoul, Republic of Korea) and approved on 30 November 2022 by the Ministry of Food and Drug Safety for clinical use in South Korea (product name: Envlo Tablet) [1,5]. In Phase I clinical trials, enavogliflozin showed rapid absorption with a peak plasma concentration occurring 1–3 h post-administration and a long terminal half-life of 13–29 h in single and repeated oral administrations [6]. The systemic exposure of enavogliflozin increased dose proportionally after repeated administrations in the dose range of 0.1–2.0 mg [6]. However, the fraction of urinary-excreted enavogliflozin was increased along with increasing dose after a single administration in the dose range of 0.2–5.0 mg (i.e., from 0.87% to 1.67%) [6]. In this study, the PBPK model for enavogliflozin and M1 was developed based on the reported clinical trial data in the literature (ClinicalTrials.gov: NCT03364985), whose concentration-time profiles were collected after a single or repeated dosing of enavogliflozin [6].

Certain drug metabolites may be pharmacologically and/or toxicologically meaningful, and the United States Food and Drug Administration has suggested qualifying them when they are present more than 10 percent of total drug-related exposure [7]. Enavogliflozin appears to be metabolized in the human liver microsome system and generates metabolites, such as M1 (that is, (2S,3R,4R,5S,6R)-2-(7-chloro-6-(4-cyclopropylbenzyl)-2-hydroxy-2,3-dihydrobenzofuran-4-yl)-6-(hydroxymethyl)tetrahydro-2H-pyran-3,4,5-triol) and M2 (that is, (2S,3R,4R,5S,6R)-2-(7-chloro-6-(4-(1-hydroxycyclopropyl)benzyl)-2,3-dihydrobenzofuran-4-yl)-6-(hydroxymethyl)tetrahydro-2H-pyran-3,4,5-triol) [8]. In a previous clinical trial, the metabolic ratio of M1 was estimated between 0.20 and 0.25 after daily oral administration of 0.1 to 2.0 mg enavogliflozin in humans [6]. In this study, physiologically based pharmacokinetic (PBPK) models for enavogliflozin and M1 were developed and validated using the published concentration–time profiles of the compounds in humans.

Enavogliflozin is believed to be a treatment option for patients with diabetes who have a high chance of suffering from hepatic impairment and nephrotic syndrome [9]. PBPK models can rationally predict concentration–time profiles compared to conventional compartment models for first-in-human, special populations, drug–drug interactions, and pathophysiological situations [10,11,12,13,14]. As quantitative measures of physiological changes have been reported in the patients with hepatic impairment [12], the developed PBPK model could be used in pharmacokinetic predictions for the special populations in further studies.

The objective of this study was to develop and validate a PBPK model for orally administered enavogliflozin in humans.

## 2. Materials and Methods

### 2.1. Model Structure

**Figure 1:**
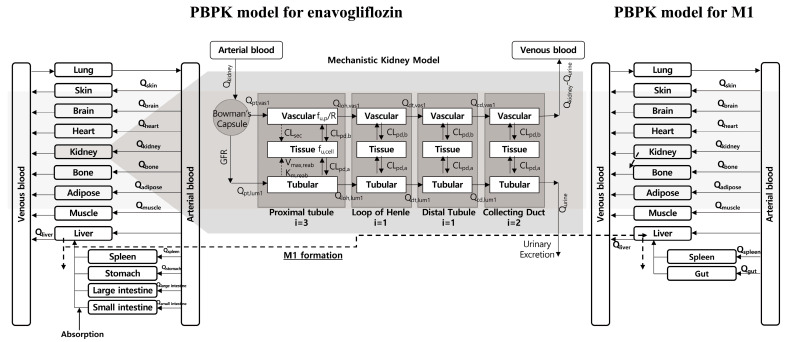
PBPK model scheme for enavogliflozin and metabolite M1 in humans after oral administration of enavogliflozin.

To predict compound concentrations, PBPK models for enavogliflozin and metabolite M1 were developed. Whole-body PBPK models consisted of 15 and 13 compartments for enavogliflozin and M1, respectively, including the arterial/venous blood pool and the major tissues (Figure 1). For the kidney compartments, mechanistically arranged kidney sub-compartments were assumed based on the anatomical structure of the tissues, as described in the mechanistic kidney model section below. The anatomical weight and blood flow rates of the tissues were obtained from published data (Davies and Morris, 1993; Brown et al., 1997) [13,15,16] and are summarized in Table 1.

Numerical simulations of the PBPK models were performed using Berkeley Madonna software version 10.4.2 (Berkeley Madonna, Inc., Albany, CA, USA). In the present study, the fourth-order Runge–Kutta method was used for numerical integration. Because there were two PBPK models for the two compounds (enavogliflozin and M1), the compound for parameters were specified in a form of subscripts if it is needed.

### 2.2. Absorption

A first-order kinetics was used to describe the absorption of enavogliflozin in humans. The differential equation for the enteral compartment (i.e., the absorption compartment) is:

(1) $$\frac{dX_{a}}{dt}=-K_{a}\cdot X_{a,enavo}$$

where $X_{a,enavo}$ is the amount of enavogliflozin remaining in the absorption compartment (e.g., the intestinal lumen), and $K_{a}$ is the first-order absorption rate constant. The initial amount of enavogliflozin in the absorption compartment was set to be the product of $F_{a}$ (i.e., fraction absorbed, predicted as 86.6%), $F_{g}$ (i.e., not metabolized fraction in the gastrointestinal tract, assumed as 1), and the administered dose.

Drug permeability in the human jejunum was predicted by the empirical relationship between Caco-2 permeability ($P_{app}$; 10^−6^ cm/s) in vitro and jejunum effective permeability ($P_{eff}$; 10^−4^ cm/s) in vivo using the following empirical correlation [17]:

(2) $$\log P_{eff}=0.4926\times \log P_{app}-0.1454$$

In this study, the experimentally determined Caco-2 $P_{app}$ value of enavogliflozin (2.4 × 10^−6^ cm/s) was scaled using propranolol as a reference compound (i.e., $P_{app}$ was multiplied by a scaling factor of 2.8) to fit the literature’s values [17]. Subsequently, the fraction of the drug absorbed ($F_{a}$) was predicted using the relationship between the effective permeability ($P_{eff}$) using the following equation and the reported value in the literature [18]:

(3) $$F_{a}=1-e^{-2\cdot \frac{T_{res}}{r}\cdot P_{eff}}$$

where $T_{res}$ is the transit time in the small intestine (~3 h) [18] and r is the radius of the small intestine. The first-order absorption rate ($K_{a}$) was also predicted by its relationship with the human jejunum effective permeability coefficient (i.e., $K_{a}=2\cdot P_{eff}/r$), in which the human intestinal tract is assumed to be a cylindrical tube.

### 2.3. Distribution

For enavogliflozin and M1, a perfusion-limited distribution was assumed for all tissue compartments, except the liver model of enavogliflozin. For tissues following perfusion-limited distribution, the differential equation for non-eliminating organs (i.e., tissues except for the liver and kidney) was as follows:

(4) $$V_{T}\cdot \frac{dC_{T}}{dt}=Q_{T}\cdot \left(C_{art}-\frac{C_{T}\cdot R}{K_{p}}\right)$$

where $V_{T}$ is the anatomical volume of the tissue compartment, $C_{T}$ and $C_{art}$ are the enavogliflozin or M1 concentrations in the tissue and arterial blood compartments, respectively, $Q_{T}$ is the blood flow to the tissue, R is the blood-to-plasma partition coefficient, and $K_{p}$ is the tissue-to-plasma partition coefficient. For the lung compartment, the input blood flow was from the venous blood pool, and $C_{art}$ in the equation above was substituted by the compound concentration in the venous blood pool [i.e., $V_{LU}\cdot \frac{dC_{LU}}{dt}=Q_{LU}\cdot \left(C_{ven}-\frac{C_{LU}\cdot R}{K_{p,LU}}\right)$].

For the liver compartment, the enavogliflozin concentration in the input blood flow could be estimated using the following equation:

(5) $$Q_{LI}\cdot C_{in,enavo}=K_{a}\cdot X_{a,enavo}+\left(Q_{LI}-Q_{ST}-Q_{SP}-Q_{Sm,IN}-Q_{La,IN}\right)\cdot C_{art,enavo}+Q_{ST}\cdot \frac{C_{ST,enavo}\cdot R_{enavo}}{K_{p,ST,enavo}}+Q_{SP}\cdot \frac{C_{SP,enavo}\cdot R_{enavo}}{K_{p,SP,enavo}}+Q_{Sm,IN}\;\cdot \frac{C_{Sm,IN,enavo}\cdot R_{enavo}}{K_{p,Sm,IN,enavo}}+Q_{La,IN}\cdot \frac{C_{La,IN,enavo}\cdot R_{enavo}}{K_{p,La,IN,enavo}}$$

where $C_{ST,enavo}$, $C_{SP,enavo}$, $C_{Sm,IN,enavo}$, and $C_{La,IN,enavo}$ are the enavogliflozin concentrations in the stomach, spleen, and small and large intestine, respectively; $Q_{LI}$, $Q_{ST}$, $Q_{SP}$, $Q_{Sm,IN}$, and $Q_{La,IN}$ are the blood flow to the liver, stomach, spleen, and small and large intestine, respectively; and $K_{p,LI,enavo}$, $K_{p,ST,enavo}$, $K_{p,SP,enavo}$, $K_{p,Sm,IN,enavo}$, and $K_{p,La,IN,enavo}$ are the tissue-to-plasma partition coefficients for the liver, stomach, spleen, and small and large intestine for enavogliflozin, respectively.

In the in vitro non-clinical study, enavogliflozin appeared to be a substrate for OATP1B1 and OATP1B3 transporters, and the respective $K_{m}$ values were 39.6 and 50.6 μmol/L in the transiently transporter-expressing HEK293 cells (Corning, Tewksbury, MA, USA). Permeability- and perfusion-limited models were integrated for the liver in the enavogliflozin model to describe the contribution of hepatic uptake transporters (e.g., OATP1B1 and OATP1B3). The integrated model, referred to as Model 1, is called the TUBE model by Jeong et al. [19,20] and is a generalized form of the extended clearance concept [21]. Passive diffusion was estimated from the published correlation between physicochemical properties and passive permeability [22]. Even though, there was some clue for the active transport into the liver, specific value for the active permeability term was optimized based on the clinical trial data [6]. The observed concentration-time profiles after a single oral administration of 1 mg enavogliflozin to humans [6] using nonlinear regression method incorporated in the Curve Fit function of Berkeley Madonna software version 10.4.2 after assuming a linear kinetics in the uptake process. The effective surface area was allometrically scaled from the literature (Table S1) [20,23]. The distribution fraction for enavogliflozin to the liver ($f_{d,LI,enavo}$) was calculated as below:

(6) $$f_{d,LI,enavo}=1-e^{\left[\frac{-{PS}_{inf,enavo}\cdot f_{up,enavo}}{R_{enavo}\cdot Q_{LI}}\right]}$$

where ${PS}_{inf,enavo}$ is the uptake clearance from the extracellular compartment in the liver into hepatocytes for enavogliflozin. Consequently, the enavogliflozin in the liver compartment was calculated using the following equation:

(7) $$\begin{array}{cc} V_{LI}\cdot \frac{dC_{LI,enavo}}{dt}= & Q_{LI}\\ & \cdot \{C_{in,enavo}\cdot \left(1-f_{d,LI,enavo}\right)+\frac{{PS}_{eff,enavo}}{{PS}_{inf,enavo}}\cdot \frac{f_{u,LI,enavo}}{f_{up,enavo}}\cdot R_{enavo}\\ & \cdot C_{LI,enavo}\cdot f_{d,LI,enavo}-CL_{u,int,enavo}\cdot f_{u,LI,enavo}\cdot C_{LI,enavo}\} \end{array}$$

where $V_{LI}$ is the anatomical volume of the liver and $CL_{u,int,enavo}$ is the intrinsic clearance of enavogliflozin in the liver compartment that was estimated from in vitro microsomal clearance using microsomal protein per gram of liver (e.g., 40 mg protein/g liver) and the liver weight [16,24]. $f_{u,LI,enavo}$ is the unbound fraction of enavogliflozin in the hepatocyte compartments estimated from the predicted liver-to-plasma partition coefficient using Rodgers and coworker’s method considering binding terms [25,26], and ${PS}_{eff,enavo}$ is the distributional clearance from the liver cells to extracellular space in the liver for enavogliflozin that consists of passive permeability.

For M1, the compound concentration in the input blood pool was estimated as follows:

(8) $$Q_{LI}\cdot C_{in,M1}=\left(Q_{LI}-Q_{Gut}-Q_{SP}\right)\cdot C_{art,M1}+Q_{Gut}\cdot \frac{C_{Gut,M1}\cdot R_{M1}}{K_{p,Gut,M1}}+Q_{SP}\cdot \frac{C_{SP,M1}\cdot R_{M1}}{K_{p,SP,M1}}+CL_{u,int,enavo}\cdot f_{u,LI,enavo}\cdot C_{LI,enavo}\;\cdot f_{m,M1}\cdot \frac{{MW}_{M1}}{{MW}_{enavo}}$$

where $C_{in,M1}$, $C_{art,M1}$, $C_{Gut,M1}$, and $C_{SP,M1}$ are the M1 concentrations in the input blood, arterial blood pool, gut, and spleen compartment, respectively; $K_{p,Gut,M1}$ and $K_{p,SP,M1}$ are the tissue-to-plasma partition coefficients for M1 in the gut and spleen compartments, respectively; $f_{m,M1}$ is the fraction of metabolism which forms M1 from enavogliflozin as a result of the hepatic metabolism of enavogliflozin; and ${MW}_{enavo}$ and ${MW}_{M1}$ are the molar masses of enavogliflozin and M1, respectively. Molar masses were necessary as the calculation was performed in the gram-based unit (i.e., not in mol unit).

For the venous blood compartment, the following equation was used:

(9) $$V_{ven}\cdot \frac{dC_{ven}}{dt}=Q_{AD}\cdot \frac{C_{AD}\cdot R}{K_{p,AD}}+Q_{BR}\cdot \frac{C_{BR}\cdot R}{K_{p,BR}}+Q_{HE}\cdot \frac{C_{HE}\cdot R}{K_{p,HE}}+Q_{KI,out}\cdot \frac{C_{KI}\cdot R}{K_{p,KI}}+Q_{LI}\cdot \frac{C_{LI}\cdot R}{K_{p,LI}}+Q_{SK}\cdot \frac{C_{Sk}\cdot R}{K_{p,SK}}+Q_{BO}\;\cdot \frac{C_{BO}\cdot R}{K_{p,BO}}+Q_{MU}\cdot \frac{C_{MU}\cdot R}{K_{p,MU}}+Q_{RE}\cdot C_{art}-Q_{CO}{\cdot C}_{ven}$$

where $V_{ven}$ is the anatomical volume of venous blood; $C_{AD}$, $C_{BR}$, $C_{HE}$, $C_{KI}$, $C_{LI}$, $C_{Sk}$, $C_{BO}$, $C_{MU}$, and $C_{ven}$ are enavogliflozin or M1 concentrations in the adipose, brain, heart, kidney, liver, skin, bone, muscle, and venous blood compartments, respectively; $Q_{AD}$, $Q_{BR}$, $Q_{HE}$, $Q_{KI}$, $Q_{LI}$, $Q_{SK}$, $Q_{BO}$, $Q_{MU}$, and $Q_{RE}$ are the blood flows to the adrenal gland, adipose, brain, heart, kidney, liver, skin, bone, muscle, and the residual blood flow, respectively; $Q_{CO}$ is the cardiac output; and $K_{p,AD}$, $K_{p,BR}$, $K_{p,HE}$, $K_{p,KI}$, $K_{p,LI}$, $K_{p,SK}$, $K_{p,BO}$, and $K_{p,MU}$ are the tissue-to-plasma partition coefficients of adipose, brain, heart, kidney, liver, skin, bone, and muscle, respectively, for enavoglifozin or M1. In the case of the kidney, the outflow of blood from the kidney was adjusted by the filtrate loss via the mechanistic kidney model (i.e., $Q_{KI,out}=Q_{KI,in}-Q_{urine}$).

The tissue-to-plasma partition coefficients ($K_{p}$) of the tissue compartments were predicted for enavogliflozin and M1 according to the method described by Rodgers and Rowland [25,26]. In this study, the $K_{p}$ value of adipose tissue was predicted using the octanol-to-water partition coefficient for enavogliflozin (logP_o:w_) instead of the olive oil-to-water partition coefficient (logP_vo:w_) [25,26], as it provided a better fit to the observed data. The steady-state tissue-to-plasma concentration ratios ($K_{p,ss}$) were predicted using the predicted $K_{p}$ ratios and extraction ratios for the liver (that is, Kp,ss=Kp·1−ERfd, where ER is the extraction ratio, and $f_{d}$ is the distributional fraction into the tissue [19]). Using the anatomical tissue volumes in Table 1 and the predicted $K_{p,ss}$ values, the calculation of $V_{ss}$ was conducted using the following equation [27,28]:

(10) $$V_{ss}=V_{p}+V_{rbc}\cdot EP+\sum V_{T,i}K_{p,ss,i}$$

where $V_{p}$ and $V_{rbc}$ are the volumes of plasma and blood cells, respectively, and EP is the blood cells-to-plasma partition coefficient. EP was calculated as follows [28]:

(11) EP=1+(R−1)/Hct

where Hct is the hematocrit (0.45) and R is the blood-to-plasma partition coefficient.

The unbound fraction of enavogliflozin in the plasma ($f_{up,enavo}$) was determined from the results of an experiment on 1 mg/mL enavogliflozin in human blood plasma using a Rapid Equilibrium Dialysis kit (Thermo Fisher Scientific, Waltham, MA, USA) after 4 h of incubation at 37 °C. The $f_{up}$ for M1 ($f_{up,M1}$) was predicted by a published model (accessible at https://drumap.nibiohn.go.jp/; accessed on 3 January 2023), which was trained using a large dataset [29]. The blood-to-plasma partition coefficients (R) of enavogliflozin and M1 were predicted using ADMET Predictor software version 10.4 (Simulation Plus, Inc., Lancaster, CA, USA).

Albumin was assumed to be the binding protein for enavogliflozin and M1 [12] because compounds are slightly acidic. The assumption was needed for the prediction of unbound fraction and blood-to-plasma partition coefficient in the patients with impaired liver [30]. The tissue bindings were adjusted using altered albumin concentrations and hematocrit values in the pathological condition. Tissue-to-plasma partition coefficients were adjusted using the altered unbound fraction in plasma, assuming that the unbound fractions in the tissues were not affected by the disease condition.

### 2.4. Elimination

In this study, elimination in the PBPK model consisted of both hepatic and renal clearances. Hepatic metabolism was in vitro–in vivo extrapolated using the results of microsomal stability. The microsomal stability of enavogliflozin was observed temporally in a 0.25 mg microsomal protein/mL suspension with NADPH either with or without UDPGA. The unbound fraction of enavogliflozin and M1 in the microsomal suspension was predicted by ADMET Predictor software version 10.4.0.5 64-bit edition (Simulation Plus, Inc., Lancaster, CA, USA) for the 1 mg protein/mL condition and adjusted to the real experimental condition (0.25 mg protein/mL; i.e., fu,undiluted=1D1fu,diluted−1+1D, where D is the dilution factor in the system [31,32]). As only 3% of a dose of the unchanged form was excreted as bile in bile duct-cannulated rats after oral administration of ^14^C-enavogliflozin [8], the contribution of bile elimination was neglected in the PBPK model for enavogliflozin.

The amount excreted through the renal route occupied 0.87–1.67% of the total dose of enavogliflozin after a single dose in a clinical trial [6]. Thus, the contribution of renal excretion to the total elimination of enavogliflozin appeared to be minor. However, the kidney is thought to be a target tissue for enavogliflozin, and the fraction of renal excretion ($f_{e}$) seems to increase with increasing doses [6]. To estimate the relative change in enavogliflozin concentration in the kidney, a mechanistically arranged kidney model was used. Non-linear reabsorption was incorporated into the kidney model based on the mechanistically arranged model [33,34,35].

M1 is a metabolite of enavogliflozin. In a previous study, enavogliflozin was not significantly eliminated in the intestinal microsomal suspension. Briefly, 95.7 ± 15.3% or 111.1 ± 26.2% of the initial enavogliflozin remained after incubating 2 μM enavogliflozin for 120 min at 37 °C in human intestinal microsomal suspensions with or without UDPGA in the system, respectively (n = 3), and there was no statistical difference among the measured % remaining in 5, 15, 30, 45, 60, and 120 min after incubation of enavogliflozin started in the intestinal microsome suspension (*p* > 0.05, one-way ANOVA). As enavogliflozin seemed to be metabolized primarily in the liver, the site of M1 formation was assumed to be the liver. The proportion of M1 formation ($f_{m,M1}$) was estimated from the ratio between the elimination clearance of enavogliflozin in human liver microsomes and the formation clearance of M1 in human liver microsomes. The formation rate of M1 was obtained from human liver microsomes and recombinant enzymes in the literature [8]. The rate of M1 formation in recombinant enzymes was scaled by the P450 abundance (i.e., 142 pmol/mg protein for CYP3A4 and 14 pmol/mg protein for CYP2C19) and intersystem extrapolation factors (ISEF) for CYP3A4 and 2C19 from the literature [36]. The mean ISEFs for CYP3A4 and 2C19 were 0.154 and 0.248, respectively, based on the intrinsic clearance of the reference compounds in the literature (i.e., midazolam, testosterone and nifedipine for CYP3A4, and S-mephenytoin for CYP2C19) [36]. The calculated formation fractions of M1 were estimated from both the recombinant enzyme and the human liver microsome system and compared. The formation in the PBPK model incorporated the results from the two systems (i.e., microsomes, recombinant enzymes). The formation rate of M1 and M2 was calculated using the Michalis–Menten equation (${CL}_{u,HLM,(M1\;or\;M2)}=\frac{v_{max,HLM,(M1\;or\;M2)}}{K_{m,HLM,(M1\;or\;M2)}\cdot f_{u,mic,enavo}+f_{u,LI,enavo}\cdot C_{LI,enavo}}$, where $C_{LI,enavo}$ and $f_{u,LI,enavo}$ are the enavogliflozin concentration and unbound fraction in the liver, and $v_{max,HLM,(M1\;or\;M2)}$ and $K_{m,HLM,(M1\;or\;M2)}$ are the maximum rate and Michaelis–Menten constants for M1 or M2 formation in the human liver microsomes) and reported constants [8]. The fraction of M1 and M2 formation ($f_{m,M1}$ and $f_{m,M2}$) was predicted dynamically, as follows:

(12) $$f_{m,(M1\;or\;M2)}=\frac{{CL}_{u,HLM,(M1\;or\;M2)}}{{CL}_{u,int,mic,enavo}}$$

where $CL_{u,int,mic,enavo}$ is the unbound intrinsic clearance of enavogliflozin elimination in the liver microsome. (e.g., $f_{m,M1}=\frac{\left(\frac{v_{max,HLM,M1}}{K_{m,HLM,M1}\cdot f_{u,mic,enavo}+f_{u,LI,enavo}\cdot C_{LI,enavo}}\right)}{{CL}_{u,int,mic,enavo}}$). Since the unbound fraction in the recombinant enzyme system was not reported for enavogliflozin, the absolute formation clearance could not be converted from the recombinant enzyme to the microsome system. The contribution of isozymes were estimated using the scaled results of recombinant enzyme assay for the prediction of altered formation rate in the pathophysiological condition (e.g., $f_{m,3A4}=f_{m,M1}\cdot \frac{\frac{v_{max,3A4,M1}}{K_{m,3A4,M1}}}{\frac{v_{max,3A4,M1}}{K_{m,3A4,M1}}+\frac{v_{max,2C19,M1}}{K_{m,2C19,M1}}}+f_{m,M2}\cdot \frac{\frac{v_{max,3A4,M2}}{K_{m,3A4,M2}}}{\frac{v_{max,3A4,M2}}{K_{m,3A4,M2}}+\frac{v_{max,2C19,M2}}{K_{m,2C19,M2}}}$).

Altered elimination of enavogliflozin was predicted based on the reported model parameters with liver cirrhosis in the literature [12]. The changed activity of CYP2C19 enzyme is calculated from the reported plasma clearance of mephenytoin and formation of 4-hydroxymephenytoin [37], and functional liver mass [12] in the patients with liver cirrhosis. There were no accessible observations about M1 elimination. Metabolic rate and renal clearance for M1 were predicted using the published methods (accessible at https://drumap.nibiohn.go.jp/; accessed on 3 January 2023) [38,39]. The predicted microsomal intrinsic clearance for M1 was assumed to be unbound one, as the literature handled the intrinsic clearance with unbound concentration (that is, Equation (2) of the literature: ${CL}_{h}=Q_{h}\cdot \frac{f_{up}\cdot {CL}_{u,int}}{Q_{h}+f_{up}\cdot {CL}_{u,int}}$) [38].

### 2.5. Mechanistic Kidney Model

A mechanistic kidney model for enavogliflozin was developed to predict its non-linear urinary excretion mainly based on the model structures and values with slight changes reported by Pletz et al. [33] and Scotcher et al. [34,35]. Because the mechanistic kidney model in this study was utilized only for the enavogliflozin (Figure 1), the compound name was not specified on the name of model parameters for the kidney model. The mechanistic kidney model consists of 21 compartments, reflecting the physiological segmentations of the kidney [33,34,35]. The kidney is divided into four major segments (i.e., proximal tubule, loop of Henle, distal tubule, and collecting duct), which are further divided into subsegments (i.e., three subsegments for proximal tubule, one subsegment for the loop of Henle and distal tubule each, and two subsegments for the collecting duct). Each subsegment is divided into three compartments: tubular lumen, cellular compartment, and vascular blood section (Figure 1). The volumes and tubular flow rates of each segment of the kidney were obtained from the literature [33,34,35] and are listed in Tables S2 and S3. The unbound fraction in the kidney cells for enavogliflozin ($f_{u,cell}$) was estimated from the predicted tissue-to-plasma partition coefficient using Rodgers and coworker’s method [25,26,30], which incorporated various binding terms. In the kidney model, the Michaelis–Menten kinetics model was assumed for non-linear reabsorption, which describes the observed non-linearity in the fraction of renal excretion ($f_{e}$). The constants for the Michaelis–Menten equation (i.e., $K_{m,reab}$ and $v_{max,reab}$) and a secretionary clearance (${CL}_{sec}$) were optimized using the observed enavogliflozin amount in urine after a single oral administration of 0.2–5 mg enavogliflozin in humans (training set), and validated using the enavogliflozin concentration in urine after repeated administration of enavogliflozin in humans (validation set). A detailed description of the kidney sub-compartments is provided in Appendix A along with the mass-balanced equations (Equations (A1)–(A14)).

### 2.6. Modelling Strategies

The results from the clinical trials were obtained from Daewoong Pharmaceutical Company and have already been published in an academic journal (ClinicalTrials.gov: NCT03364985) [6]. The observed concentration-time profiles were divided into two groups, the training and validation sets. During the model refinement process, results from a single-dose oral administration study of enavogliflozin ranging from 0.2 to 5 mg were used. For model validation, a repeated administration study for 15 days with doses ranging from 0.3 to 1 mg/day was used. There was no training set for the M1 model, and the M1 concentration-time profiles after repeated administration were utilized for model validation.

The proposed PBPK model was validated by comparing the AUC_inf_, AUC_τ_ (i.e., area under the plasma concentration–time curve from time zero to infinity at the single dosing regimen and a dosing period at the repeated dosing regimen, respectively), and C_max_ (i.e., the maximum plasma concentration) values from the simulations to those from the clinical data of repeated administration studies. In the present study, the fold differences of the resulting AUC ratios (AUC_pred_:AUC_obs_) and C_max_ ratios (C_max,pred_:C_max,obs_) within a factor of two (0.5–2) were considered adequate for model performance estimation [13]. In the case of enavogliflozin in urine, model performance was assessed based on the ratio between the simulated and observed excreted amounts at the last sampling time after a single administration. The mean amounts and concentrations were used as the observed values for the model validation.

### 2.7. Staticstics and Data Analysis

Statistical differences between two groups were determined using Student’s *t*-test, and one-way ANOVA was used for multiple comparisons. In the present study, data were expressed as means ± standard deviation (S.D.), and *p*-values < 0.05 denoted statistical significance.

Standard non-compartmental analysis was performed using WinNonlin software version 8.1 (Pharsight Corporation, Mountain View, CA, USA) and the web-based Blueberry service (accessible at https://pk-square.com; accessed on 3 January 2023). Microsoft Excel software version 2211 (Microsoft Corporation, Redmond, WA, USA) was used for the unpaired t-tests and visualization of the simulation. GraphPad Prism software version 9.5.0 (GraphPad Software, San Diego, CA, USA) was used to visualize the simulations. Web-based Chemicalize service (ChemAxon Kft., Budapest, Hungary; accessible at https://chemicalize.com/; accessed on 3 January 2023) was used to obtain several physicochemical properties.

## 3. Results

### 3.1. Optimization of the PBPK Model

The input parameters for the PBPK model were derived from in silico and in vitro studies and are summarized in Table 2. The kinetic parameters involved in absorption were derived from the Caco-2 permeability of enavogliflozin. Enavogliflozin was predicted to be rapidly absorbed ($K_{a}$ value of 0.764 h^−1^) with good permeability through intestinal membranes ($F_{a}$ value of 0.866). The unbound fraction in plasma ($f_{up}$) was observed as 0.015 ± 0.002 for enavogliflozin and predicted as 0.080 for the metabolite M1 [29]. The extent of distribution was derived from the in silico prediction of tissue partition coefficients ($K_{p}$). The volumes of distribution at steady state ($V_{ss}$) were predicted to be 1.44 L/kg for enavogliflozin and 0.431 L/kg for the metabolite M1.

**Figure 2:**
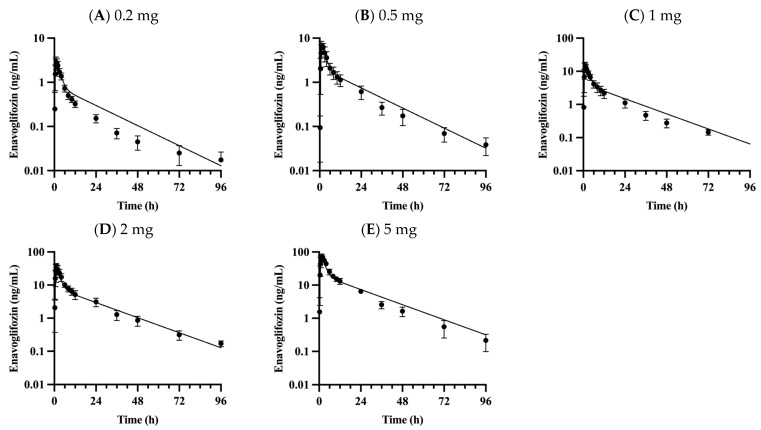
Observed and simulated plasma concentration profiles for enavogliflozin in humans following single doses of 0.2 (**A**), 0.5 (**B**), 1 (**C**), 2 (**D**), and 5 mg (**E**). Solid lines represent the optimized simulations, and closed circles (●) represent observed data. Data are expressed as means ± S.D. from eight to ten healthy volunteers.

Preclinical data suggest that enavogliflozin is primarily eliminated by hepatic pathways, including metabolism and bile excretion. Indeed, phase I clinical data reports that renal excretion of enavogliflozin is negligible (less than 2.5%) in humans [6]. Therefore, the predicted metabolic clearance was assumed to be the only intrinsic clearance in the liver. The unbound fraction of enavogliflozin in microsomal suspension was predicted to be 0.577 in 1 mg protein/mL using ADMET Predictor software version 10.4.0.5 and adjusted to 0.845 in the 0.25 mg protein/mL condition after considering the dilution factor. The intrinsic clearance was obtained from an in vitro human microsomal stability assay (13.5 μL/min/mg protein), and the unbound clearance was calculated as 16.0 μL/min/mg protein for enavogliflozin. The unbound intrinsic clearance was incorporated into the model with physiological scalars (e.g., milligram protein per gram of the liver and liver weight in humans) [16,24]. There was no statistically significant difference in the intrinsic clearance of enavogliflozin with or without UDPGA in the human microsomal suspension based on the t-test (*p* = 0.329). The active uptake clearance (${PS}_{u,inf,act,enavo}$) was optimized as 9.73 L/h/kg using the observed concentration–time profiles after a single oral administration of 0.2 to 5 mg enavogliflozin orally in humans (training set; Figure 2).

Renal excretion was predicted using a mechanistic kidney model that incorporated a non-linear reabsorption term for enavogliflozin in humans. The model parameters for the kidney were obtained after non-linear regression using the observed cumulative renal excretion after a single administration of enavogliflozin in humans. The obtained parameters were 0.0845 ng/mL, 305 ng/h and 3.39 L/h for $K_{m,reab}$, $v_{max,reab}$, and the secretionary clearance (${CL}_{sec}$), respectively.

Among the metabolites of enavogliflozin, the fractions for M1 and M2 formation ($f_{m,(M1\;or\;M2)}$) were estimated as 48.4% and 10.4% after comparing the intrinsic elimination clearance (13.5 μL/min/mg protein) for enavogliflozin and the formation clearance for M1 and M2 (6.53 μL/min/mg protein for M1 and 1.41 µL/min/mg protein for M2) in human microsomal suspension from the literature [8]. The $f_{m}$ for M1 and M2 was also calculated based on the results of M1 and M2 formation rate in the recombinant enzymes. As the ISEF-CL_int_ and P450 abundance could be obtained from the literature [8,36], the results between the different isozymes (i.e., CYP3A4 and CYP2C19) were compared. The contribution of CYP3A4 for M1 and M2 formation were 94.4% and 64.6%, respectively, which was comparable to the results using specific antibodies in the literature [8]. Those of CYP2C19 for M1 and M2 were 5.62% and 35.3%, respectively. Thus, CYP3A4 and CYP2C19 appeared to cover 52.4% and 6.42% of enavogliflozin metabolism in the liver.

For the PBPK model of M1, the compound’s elimination rate should be assigned. However, there were no accessible in vitro and in vivo experimental results for the metabolite M1. The unbound intrinsic clearance for M1 was predicted in silico to be 30.542 μL/min/mg protein [38]. The renal clearance was predicted in silico to be 42.27 mL/h/kg by the published method [39].

### 3.2. Validation of the PBPK Model

**Figure 3:**
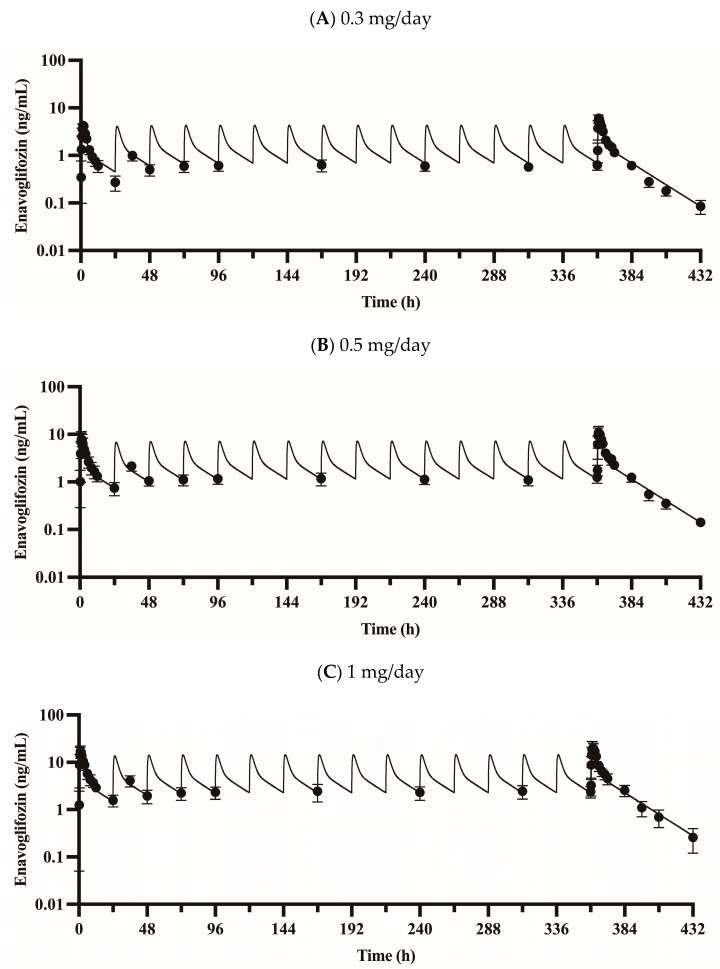
Observed and predicted plasma enavogliflozin concentration profiles in humans during repeated doses of 0.3 (**A**), 0.5 (**B**), and 1 mg enavogliflozin (**C**) administered once daily for 15 days. Solid lines represent the model prediction (i.e., simulated data), and closed circles (●) represent observed data. Data are expressed as means ± S.D. from eight healthy volunteers.

**Figure 4:**
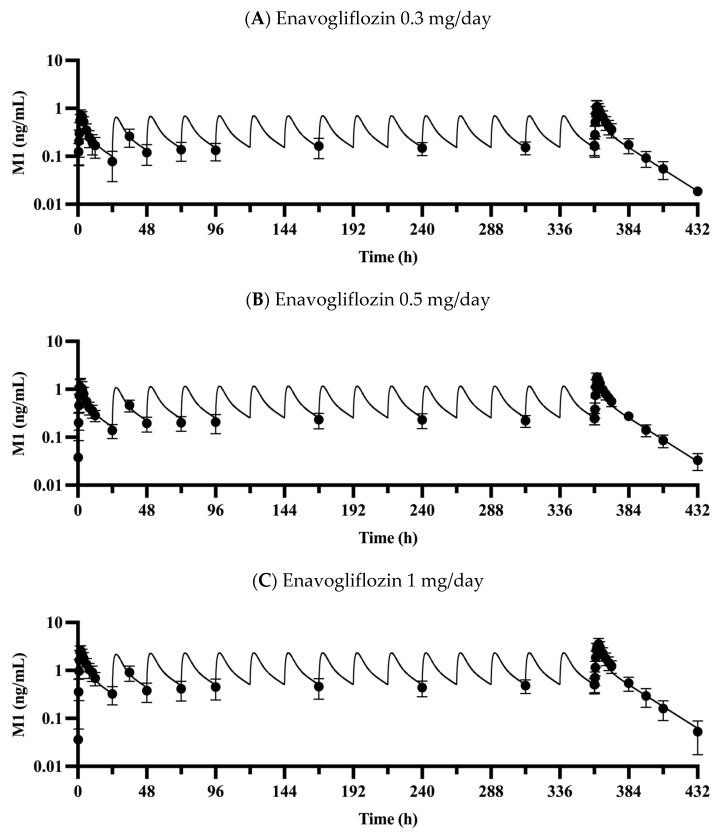
Observed and predicted plasma concentration profiles for the metabolite M1 at repeated doses of 0.3 (**A**), 0.5 (**B**), and 1 mg enavogliflozin (**C**) administered to humans once daily for 15 days. Solid lines represent the model predicted M1 concentration (i.e., simulated data), and closed circles (●) represent observed data. Data are expressed as means ± S.D. from eight healthy volunteers.

The proposed PBPK model captured the plasma concentration–time profiles of enavogliflozin (Figure 2) following a single dose (0.2–5 mg enavogliflozin) and repeated doses (i.e., the validation set of 0.3, 0.5, 1 mg/day for 15 days; Figure 3) in humans. The estimated AUC_inf_ and C_max_ ratios between simulated and observed values ranged from 0.901 to 1.25 and from 0.812 to 1.04, respectively, after the single administration of enavogliflozin (Table 3). When the proposed model was used to predict the systemic pharmacokinetics of enavogliflozin obtained from the validation dataset (Figure 3), the AUC_τ_ and C_max_ ratios for the first day of administration ranged from 0.811 to 1.05 and 0.712 to 0.869, respectively. In addition, the AUC_τ_ and C_max_ ratios for the last day of administration ranged from 0.758 to 0.880 and 0.660 to 0.727, respectively (Table 3). Furthermore, the concentration–time profiles of M1 were predicted using the developed PBPK model (Figure 4). Because there were no optimized parameters in the M1 model, there was no training set for the M1 model among the clinical data. The AUC_τ_ ratio and C_max_ ratio of M1 were predicted and compared to those of the observed parameters after repeated enavogliflozin dosing in humans (Table 4). The AUC_τ_ ratio ranged from 0.762 to 1.06, and the C_max_ ratio was between 0.641 and 0.829 for M1 in the range of repeated enavogliflozin doses. The AUC ratio and C_max_ ratio were within the two-fold error range, which was assumed to be the acceptable range of the model performance in the method section. Collectively, the PBPK models developed in this study were found to be valid according to preset criteria.

**Figure 5:**
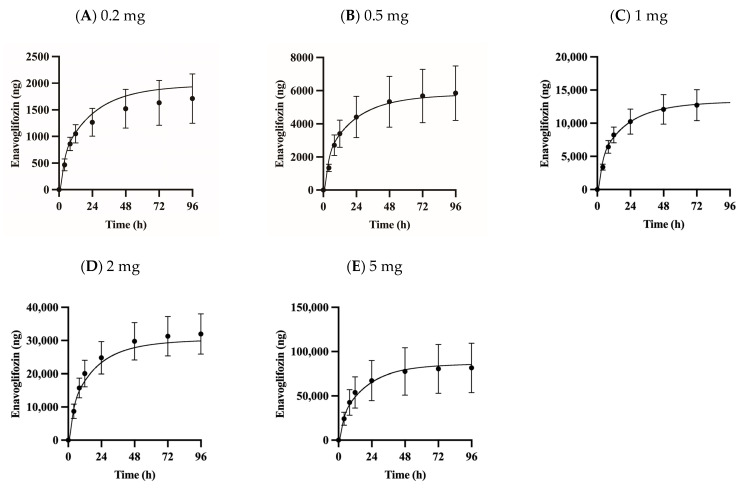
Cumulative urinary excretion profiles of enavogliflozin at single doses of 0.2 (**A**), 0.5 (**B**), 1 (**C**), 2 (**D**), and 5 mg (**E**) to humans. Solid lines represent the optimized simulations, and closed circles (●) represent observed data. Data are expressed as means ± S.D. from eight healthy volunteers.

A mechanism-based kidney model for enavogliflozin was developed using the urinary excretion profile of the drug administered within the dose ranges of 0.2–5 mg. The cumulative observed amounts of enavogliflozin in urine were 1.71 ± 0.463, 5.85 ± 1.65, 12.7 ± 2.34, 32.0 ± 6.07, and 81.6 ± 27.9 μg at the last sampling time for excretion after the single administration of 0.2, 0.5, 1, 2, and 5 mg of enavogliflozin in humans, respectively (training set) [6]. The simulated cumulative amounts of enavogliflozin excreted in urine were 1.93, 5.69, 12.8, 29.8, and 85.2 μg after the single administration of 0.2, 0.5, 1, 2, and 5 mg of enavogliflozin, respectively, at the same time point (Figure 5) [33,34,35]. The simulated amount of urinary-excreted enavogliflozin was in the two-fold range of the observed value at the last sampling time after any single dosage examined in humans.

## 4. Discussion

This was the first study for publication to simulate concentration–time profiles of enavogliflozin and its metabolite M1 based on PBPK models in humans. In this study, PBPK models for enavogliflozin and its metabolite M1 were developed and validated using in silico and in vitro data accompanied by clinical observations. The PBPK model developed in this study can be used to extend dosing regimens and predict drug–drug interactions and population-related alterations in the pharmacokinetic profiles of enavogliflozin in humans. For example, some marketed drugs require their dosing regimen to be adjusted or are not recommended for patients with hepatic or renal impairment [12,41,42,43]. Additionally, researchers involved in new drug development may need to predict the expected exposure or concentration profiles of the agent before clinical observation. The model-based prediction would have a role in that decision, especially the PBPK model, because of the physiological factors it accounts for, even though the prediction might be verified after clinical trials in the patient population.

In a previous study, M1 was reported to have metabolic ratios (i.e., AUC ratio between M1 and enavogliflozin) of 25%, 21%, and 22% for the repeated doses of 0.3, 0.5, and 1 mg/day, respectively [6]. Based on the AUC_τ_ after the last dose of repeated administration study, the predicted metabolic rates for M1 were 18.8%, 16.2%, and 16.3% for the 0.3, 0.5, and 1 mg/day doses, respectively. The model appeared to reproduce the slight difference in metabolic rates seen among those doses. For the calculation of the metabolic rate, the AUC values of enavogliflozin and M1 were converted to molar-based values (i.e., not a gram-based unit, but a mol-based unit was incorporated). Though there were Michaelis–Menten constants for those two isozymes, CYP3A4 and CYP2C19, the same contribution ratio among the dose range was assumed because of the absence of the unbound fraction in the recombinant enzyme system. The assumption may be reasonable because of the high Michalis–Menten constant for those two isozymes (Table 2). Although the metabolic fraction for the formation of M1 ($f_{m,M1}$) was estimated from the in vitro assay, the volume of distribution (i.e., estimated by predicted $K_{p}$) and elimination rate for M1 [i.e., metabolism (${CL}_{u,int,M1}$) and renal excretion (${CL}_{r,M1}$)] were predicted using in silico methods [25,26,30,38,39]. Thus, the metabolic rate could be better predicted with more robust information on the formation, distribution, metabolism, and excretion of M1 (e.g., unbound fraction of enavogliflozin in the recombinant enzyme system, cumulatively excreted amount of M1 in vivo, and microsomal stability of M1 in vitro). In a published structure-activity relationship (SAR) study, hetero-bicyclic derivatives of enavogliflozin seemed to have similar IC_50_ values for SGLT2 and showed a difference in their selectivity between SGLT1 and SGLT2 [1]. However, there were no experimental IC_50_ values for the metabolite M1 in the SAR study. Further experiments and trials may be required to refine and extend the developed PBPK model along with pharmacodynamic model.

The simulated concentrations or amounts of enavogliflozin in plasma and urine, respectively, seemed to match well with observed profile (Figure 2, Figure 3, Figure 4 and Figure 5), and the predicted pharmacokinetic parameters met the criteria (Table 3 and Table 4). Therefore, the developed PBPK model was validated using the established criteria in this study. As a model challenge, the absolute bioavailability was predicted for oral administration in humans. This predicted bioavailability ranged from 78.9% to 79.0% after a single dose of 0.2, 0.5, 1, 2 and 5 mg and at the steady-state after repeated doses of once-daily administration of 0.3, 0.5 and 1 mg enavogliflozin. Although there have been no clinical trials testing intravenous doses, the predicted oral bioavailability in humans is comparable to the reported values in the animal experiments, which were 84.5–97.2% in mice and 56.3–77.4% in rats [1,44]. Even though the absorption model had a simple structure (i.e., the first-order kinetics), the predicted exposure (AUC) and C_max_ matched to the observed values well. However, a more sophisticated model for the absorption (e.g., CAT, A-CAT, and ADAM models) might be needed to study the absorption level alteration [45,46,47,48].

In the aspects of intestinal metabolism, there were experimental results for enavogliflozin using human intestinal microsome that showed no statistically meaningful elimination (*p* > 0.05, one-way ANOVA test for each measuring time) in the intestinal microsomal suspension with NADPH only or with both NADPH and UDPGA. Briefly, the percent remaining after incubation were 97.2 ± 24.2%, 109.8 ± 26.2%, 117.5 ± 27.8%, 96.4 ± 7.2%, 101.2 ± 22.9%, and 111.1 ± 26.2% for 5, 15, 30, 45, 60, and 120 min, respectively, after the initiation of incubation with NADPH only, and 106.7 ± 3.8%, 110.2 ± 5.7%, 107.5 ± 10.2%, 87.2 ± 10.9%, 90.5 ± 10.1%, and 95.7 ± 15.3% for 5, 15, 30, 45, 60, and 120 min, respectively, after the incubation starts with NADPH and UDPGA. Though there was no statistical significance among the measured percent remaining, the variability of the measure was larger than the results in the liver microsomal suspension, especially in the group with NADPH only. Because CYP enzymes had a meaningful contribution to the liver metabolism, there might be unseen contributions of the drug-metabolizing enzymes in the intestine.

The elimination in the kidney can be attributed to excretion and metabolism [49]. In this study, renal elimination in the PBPK model of enavogliflozin was achieved via urinary excretion. Enavogliflozin revealed a dose-dependent increase in the fraction excreted in urine, and the kidney model for enavogliflozin incorporated nonlinearity in the reabsorption term. Additional observations for renal elimination can be incorporated into the kidney model for future studies, such as predicting the pharmacological effect of enavogliflozin based on pharmacodynamic modelling in humans. Despite this limitation, the PBPK model could simulate the cumulative amounts of urinary excretion of enavogliflozin in the preset range of error (i.e., 2-fold) after a single administration of the drug orally in humans (Figure 5).

**Figure 6:**
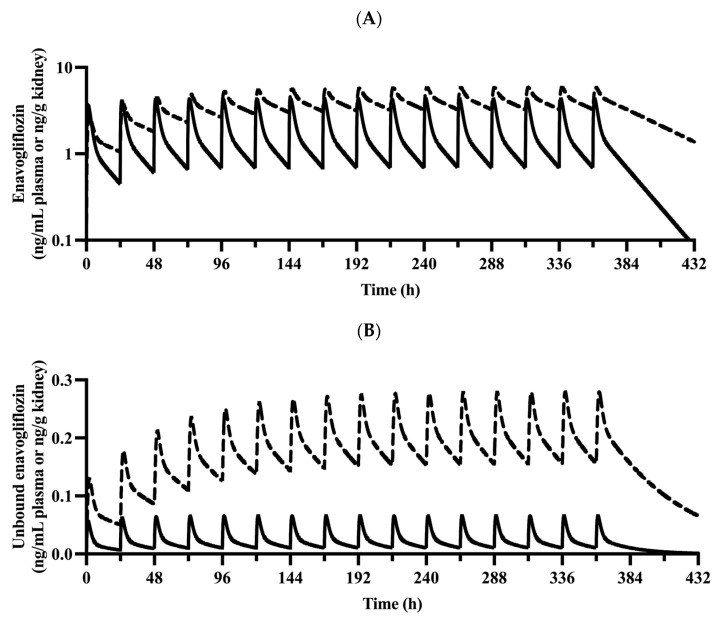
Predicted plasma and kidney total (**A**) and unbound (**B**) concentration profiles for enavogliflozin at repeated doses of 0.3 mg enavogliflozin administered to humans once daily for 15 days. Solid (―) and dashed (╍) lines represent the predicted concentration of enavogliflozin in the plasma and kidney, respectively.

Based on the model simulation (Figure 6), the half-life at the terminal phase of steady-state were 15.9 h and 38.4 h in the plasma and kidney, respectively. The difference in half-life could be linked to the higher exposure of enavogliflozin in the kidney (i.e., the target organ) than in the plasma. The AUC_τ_ for enavogliflozin in the kidney was predicted by the PBPK model as 100 ng∙h/g tissue at the steady state after 0.3 mg once daily dosing of enavogliflozin in humans, and the total and unbound kidney-to-plasma partition coefficients for enavogliflozin (i.e., $K_{p,ss,KI,enavo}$ and $K_{p,uu,ss,KI,enavo}$, respectively) were predicted to be 2.67 and 8.49, respectively (Figure 6). The targeted exposure may be helpful in expanding the therapeutic window of enavogliflozin. Using the predicted unbound AUC in the kidney, the averaged unbound concentration of enavogliflozin at a steady state in the kidney ($C_{u,avg,ss,KI,enavo}$) was calculated after 0.3 mg/day oral dosing of enavogliflozin as 0.446 nmol/L (0.199 ng/mL), which was comparable to the reported IC_50_ in the previous SAR study (i.e., 0.46 nmol/L for SGLT2) [1]. Though the kidney model in this study could describe and predict in the expected range, there might be needs in the future to incorporate more mechanisms. For example, the kidney model in this study have multiple sub-compartments and physiological flows, but there are still missing physiologies, including bypass of blood flow and pH differences in the sub-compartments, which is included in the commercial model of Simcyp software (MechKiM) [50].

PBPK modelling is useful in a predictive study, including first-in-human dose prediction, drug–drug interaction, pediatrics, geriatrics, altered physiologies, and different ethnicities [10,11,12,14,24]. As enavogliflozin is mainly eliminated by hepatic metabolism, the validated PBPK model for enavogliflozin was challenged in hepatic-impaired patients. Pathophysiological changes in the patients are described in the literature [12], and the fractional activities assumed for CYP2C19 were 0.5, 0.3, and 0.3 in the patients with Child–Pugh score A, B, and C, respectively, for the model of Andrea Edginton and Stefan Willmann [12,37]. Various isozymes are involved in the metabolism of enavogliflozin, including CYP3A4, CYP2C19, UGT1A4, UGT1A9, and UGT2B7 [8]. The primary metabolites of CYP3A4 and CYP2C19 pathways seemed to be M1 and M2, and the estimated contribution of CYP3A4 in the formation of M1 and M2 in this study was comparable to the reported results of anti-CYP3A4 antibody study in the literature [8]. The other primary metabolites were appeared to be the generated by the other drug-metabolizing enzymes, such as UGT1A4, UGT1A9, and UGT2B7 [8]. Since UGTs were not reported as a consistent change in activities for the patients with hepatic impairment [37], the rest fraction, not metabolized to M1 and M2 (41.2%), was assumed not to be affected by the activity alteration of CYP3A4 and CYP2C19 but to be affected only by the changes in the active liver mass under the liver impairments.

Among the Child–Pugh classes, a PBPK model simulation was performed. AUC_τ_ for enavogliflozin in plasma was 45.3 ng∙h/mL, 71.7 ng∙h/mL, and 103 ng∙h/mL, after orally administration of enavogliflozin 0.3 mg/day once daily in patients with Child–Pugh score of A, B, and C, respectively. The predicted AUC_τ_ for the patients with scores of A, B, and C were 121%, 191%, and 273%, respectively, compared with the prediction for healthy individuals. C_max_ were 3.76 ng/mL, 4.57 ng/mL, and 5.50 ng/mL in the patients with scores of A, B, and C, respectively, after the same dosing regimen. The predicted C_max_ values were 87.0%, 106%, and 127% compared with those predicted for healthy individuals. The predicted unbound fractions in plasma were 0.0187, 0.0222, and 0.0300 in the patients with the score A, B, and C, respectively, which were 123%, 146%, and 197% of the fraction in the plasma for healthy individuals. The predicted parameters for enavogliflozin were in the range from 80% to 125% range [51] for the patients with mild hepatic impairment (Child-Pugh score A), but the drug would have quite different pharmacokinetic characteristics in the patients with Child–Pugh scores B and C. Estimation for the M1, a major metabolite, concentration may be needed in the population for the further studies, such as dose adjustments.

## 5. Conclusions

In this study, PBPK models for enavogliflozin and M1 in humans were developed and evaluated using plasma concentration profiles from multiple clinical trials. For further study, a mechanistically arranged kidney model was developed, and the simulated unbound concentration of enavogliflozin in the kidney appeared to be relevant. The developed PBPK model may be useful for further pharmacokinetic and pharmacodynamic studies in humans.

## Figures and Tables

**Table 1:** Physiological parameters used for the whole body PBPK of enavogliflozin. The cardiac output for a representative human of 70 kg body weight was assumed to be 5200 mL/min.

Tissue	Anatomical Weight (g) ^1^	Blood Flow (mL/min) ^1^
Adipose	15,000	270
Bone	10,000	218
Brain	1400	593
Heart	329	208
Kidney	308	910
Large intestine	371	208
Liver	1800	1326
Lung	532	5200
Muscle	28,000	884
Skin	2600	260
Small intestine	637	520
Spleen	182	104
Stomach	147	52
Venous blood	3470	
Arterial blood	1730	

^1^ The relative weight and flow were obtained from the literature [13,15,16].

**Table 2:** Input parameters for PBPK modelling of enavogliflozin in humans.

Compound	Parameter		Unit	Value	Reference
Enavogliflozin	Physicochemical properties	Molar mass	g/mol	446.92	Chemicalize
		Compound type		Neutral	Chemicalize
		pKa (acidic)		12.57	Chemicalize
		logP		2.65	[40]
		$f_{up}$		0.015	Measured (See text)
		B/P ratio ($R_{enavo}$)		0.720	Predicted (See text)
		$P_{app,PAMPA}$	10^−6^ cm/s	1.74	Predicted (See text)
	Absorption	$P_{eff}$	10^−4^ cm/s	1.86	Predicted (See text)
		$K_{a}$	h^−1^	0.764	Predicted (See text)
		$F_{a}$		0.866	Predicted (See text)
	Distribution	$V_{ss}$	L/kg	1.44	Predicted [25,26]
	Elimination	${CL}_{int,enavo}$	μL/min/mg protein	13.5	Measured (See text)
		$f_{u,mic,enavo}$		0.845	Predicted (See text)
M1	Physicochemical properties	Molar mass	g/mol	462.92	Chemicalize
		Compound type		Neutral	Chemicalize
		pKa (acidic)		11.92	Chemicalize
		log P		1.982	Chemicalize
		$f_{up,M1}$		0.080	Predicted [29]
		B/P ratio ($R_{M1}$)		0.724	Predicted (See text)
	Formation	$K_{m,HLM,M1}$	nmol/mL ^1^	150.1	[8]
		$v_{max,HLM,M1}$	pmol/min/mg protein ^1^	980.3	[8]
		$K_{m,3A4,M1}$	nmol/mL ^1^	471.4	[8]
		$v_{max,3A4,M1}$	pmol/min/mg protein ^2^	631.5	Estimated (See text)
		$K_{m,2C19,M1}$	nmol/mL ^1^	156.4	[8]
		$v_{max,2C19,M1}$	pmol/min/mg protein ^2^	12.48	Estimated (See text)
	Distribution	$V_{ss}$	L/kg	0.421	Predicted [25,26]
	Elimination	${CL}_{u,int,M1}$	μL/min/mg protein	30.542	Predicted [38]
		${CL}_{r,M1}$	L/h/kg	0.04227	Predicted [39]
M2	Formation	$K_{m,HLM,M2}$	nmol/mL ^1^	58.7	[8]
		$v_{max,HLM,M2}$	pmol/min/mg protein ^1^	82.8	[8]
		$K_{m,3A4,M2}$	nmol/mL ^1^	674.5	[8]
		$v_{max,3A4,M2}$	pmol/min/mg protein ^2^	83.04	Estimated (See text)
		$K_{m,2C19,M2}$	nmol/mL ^1^	35.4	[8]
		$v_{max,2C19,M2}$	pmol/min/mg protein ^2^	2.385	Estimated (See text)

^1^ Total concentration of enavogliflozin in the microsomal suspension (i.e., the unbound fraction in the microsomal suspension was needed to estimate the unbound concentration). ^2^ The maximum rate was adjusted to the milligram microsomal protein-based value after rationally scaled from the value in the assay using recombinant enzymes.

**Table 3:** Summary of predicted and observed AUC (ng·h/mL) and C_max_ (ng/mL) ratios of enavogliflozin in single dose and repeated dose administration studies.

Dose	AUC_obs_^1, 2^ (ng·h/mL)	AUC_pred_^1^ (ng·h/mL)	AUC Ratio	C_max,obs_ (ng/mL)	C_max,pred_ (ng/mL)	C_max_ Ratio
*Training set (the single dosing study)*						
0.2 mg	20.2	25.1	1.25	2.84	2.44	0.859
0.5 mg	53.2	62.6	1.18	5.87	6.11	1.04
1 mg	110	125	1.14	14.0	12.2	0.871
2 mg	277	249	0.901	30.1	24.4	0.812
5 mg	619	621	1.00	70.7	61.0	0.863
*Validation set (1st day of the repeated dosing study)*						
0.3 mg/day	26.2	27.4	1.05	4.22	3.67	0.869
0.5 mg/day	51.5	45.6	0.886	7.86	6.11	0.778
1 mg/day	112	91.0	0.811	17.2	12.2	0.712
*Validation set (15th day of the repeated dosing study)*						
0.3 mg/day	42.7	37.6	0.880	6.04	4.32	0.714
0.5 mg/day	82.4	62.5	0.758	10.9	7.19	0.660
1 mg/day	163	125	0.762	19.7	14.4	0.727

^1^ AUC_inf_ and AUC_τ_ were calculated for the single and repeated dosing study, respectively. ^2^ AUC_obs_ was calculated using the mean concentration–time profiles for each group.

**Table 4:** Summary of predicted and observed AUC_τ_ (ng·h/mL) and C_max_ (ng/mL) ratios of M1 in the repeated dose administration studies.

Enavogliflozin Dose	AUC_obs_ * (ng·h/mL)	AUC_pred_ (ng·h/mL)	AUC Ratio	C_max,obs_ (ng/mL)	C_max,pred_ (ng/mL)	C_max_ Ratio
*Validation set (1st day of the repeated dosing study)*						
0.3 mg/day	5.73	6.05	1.06	0.725	0.564	0.779
0.5 mg/day	9.55	10.1	1.05	1.13	0.940	0.829
1 mg/day	22.3	20.1	0.901	2.56	1.88	0.734
*Validation set (15th day of the repeated dosing study)*						
0.3 mg/day	10.8	8.30	0.766	1.09	0.699	0.641
0.5 mg/day	17.0	13.8	0.811	1.80	1.16	0.646
1 mg/day	36.2	27.6	0.762	3.56	2.33	0.654

* AUC_obs_ was calculated using the mean concentration-time profiles for each group.

## Data Availability

The cited clinical trial data were published by Hwang et al. (ClinicalTrials.gov: NCT03364985) [6].

## Supplementary Materials

The following supporting information can be downloaded at: https://www.mdpi.com/article/10.3390/pharmaceutics15030942/s1, Table S1: Allometrically scaled effective surface area in humans; Table S2: Compartment volumes of the mechanistic kidney model; Table S3: Flow rates entering each compartment of the mechanistic kidney model. References [20,23,33,34,35] are cited in the supplementary materials.

## Appendix A. Kidney Sub-Compartments for Enavogliflozin

### Appendix A.1. Blood Compartments

In blood compartments, changes in drug concentrations are driven by blood flow through the segments and passive transport to the cellular compartments. For proximal tubular blood compartments, active transport to cellular compartments (i.e., secretion) was also incorporated.

Differential equations for enavogliflozin in the blood compartments were:

1. Glomerular Blood (i.e., Bowman’s Capsule)

(A1) $$\frac{d(C_{bcvas})}{dt}={(Q}_{kid}\cdot C_{ar}-\left(Q_{kid}-GFR\right)\cdot C_{bcvas}-GFR\cdot f_{ub}\cdot C_{bcvas})/V_{bcvas}$$

2. Proximal Tubular Blood

(A2) $$\frac{d(C_{ptvas,i})}{dt}=\left\{\left(Q_{kid}-GFR\right)\cdot C_{bcvas}-\left(Q_{kid}-Q_{ptlum,i+1}\right)\cdot C_{ptvas,i}-{CL}_{sec}\cdot C_{ptvas,i}\cdot f_{ub}+P_{pd}\cdot SA_{pt,i}\;\cdot \left(C_{ptc,i}\cdot f_{ucell}-C_{ptvas,i}\cdot f_{ub}\right)\right\}/V_{ptvas,i}$$

where i refers to the subsection of the proximal tubule from 1 to 3. For *i* = 2 or 3, $(Q_{kid}-Q_{ptlum,i})\cdot C_{ptvas,i-1}$ was used instead of $\left(Q_{kid}-GFR\right)\cdot C_{bcvas}$.

3. Blood at the Loop of Henle

(A3)
$$\frac{d(C_{lhvas})}{dt}=\left\{\left(Q_{kid}-Q_{lhlum}\right)\cdot C_{ptvas,3}-\left(Q_{kid}-Q_{dtlum}\right)\cdot C_{lhvas}+P_{pd}\cdot SA_{lh}\cdot \left(C_{lhc}\cdot f_{ucell}-C_{lhvas}\cdot f_{ub}\right)\right\}/V_{lhvas}$$

4. Blood at the Distal tubule

(A4)
$$\frac{d(C_{dtvas})}{dt}=\left\{\left(Q_{kid}-Q_{dtlum}\right)\cdot C_{lhvas}-\left(Q_{kid}-Q_{cdlum,1}\right)\cdot C_{dtvas}+P_{pd}\cdot SA_{dt}\;\cdot \left(C_{dtc}\cdot f_{u,cell}-C_{dtvas}\cdot f_{ub}\right)\right\}/V_{dtvas}$$

5. Blood at the Collecting Duct

(A5) $$\frac{d(C_{cdvas,i})}{dt}=\left\{\left(Q_{kid}-Q_{cdlum,i}\right)\cdot C_{dtvas}-\left(Q_{kid}-Q_{cdlum,i+1}\right)\cdot C_{cdvas,i}+P_{pb}\cdot SA_{cd,i}\;\cdot \left(C_{cdc,i}\cdot f_{u,cell}-C_{cdvas,i}\cdot f_{ub}\right)\right\}/V_{cdvas,i}$$

where i refers to the subsection of the collecting duct from 1 to 2. For *i* = 2, $\left(Q_{kid}-Q_{cdlum,i}\right)$ was multiplied with $C_{cdvas,i-1}$, and $C_{cdvas,i}$ was multiplied with $\left(Q_{kid}-Q_{urine}\right)$ instead of $\left(Q_{kid}-Q_{cdlum,i+1}\right)$.)

### Appendix A.2. Cellular Compartments

In cellular compartments, the change in drug concentrations are driven by passive diffusion between the vascular compartment and the cells and cells and luminal compartments. For proximal tubular blood compartments, active transport from cellular (i.e., secretion) and luminal (i.e., reabsorption) compartments were also incorporated.

Differential equations for enavogliflozin in the cellular compartments were:

6. Cellular Compartments at the Proximal Tubule

(A6)
$$\frac{d\left(C_{ptc,i}\right)}{dt}=\left\{{CL}_{sec}\cdot C_{ptvas,i}\cdot f_{ub}+P_{pd}\cdot SA_{pt,i}\cdot \left(C_{ptvas,i}\cdot f_{ub}-C_{ptc,i}\cdot f_{u,cell}\right)+V_{max,reab}\cdot \left(K_{m,reab}+C_{ptlum,i}\right)\;\cdot C_{ptlum,i}+P_{pd}\cdot SA_{pt,i}\cdot \left(C_{ptlum,i}-C_{ptc,i}\cdot f_{u,cell}\right)\right\}/V_{ptc,i}$$

7. The Cellular Compartment at Loop of Henle

(A7)
$$\frac{d\left(C_{lhc}\right)}{dt}=\left\{P_{pd}\cdot SA_{lh}\cdot \left(C_{lhvas}\cdot f_{ub}-C_{lhc}\cdot f_{u,cell}\right)+P_{pd}\cdot SA_{lh}\cdot \left(C_{lhlum}-C_{lhc}\cdot f_{u,cell}\right)\right\}/V_{lhc}$$

8. Cellular Compartments at the Distal Tubule

(A8)
$$\frac{d\left(C_{dtc}\right)}{dt}=\left\{P_{pd}\cdot SA_{dt}\cdot \left(C_{dtvas}\cdot f_{ub}-C_{dtc}\cdot f_{u,cell}\right)+P_{pd}\cdot SA_{dt}\cdot \left(C_{dtlum}-C_{dtc}\cdot f_{u,cell}\right)\right\}/V_{dtc}$$

9. Cellular Compartments at the Collecting Duct

(A9)
$$\frac{d\left(C_{cdc,i}\right)}{dt}=\left\{P_{pb}\cdot SA_{cd,i}\cdot \left(C_{cdvas,i}\cdot f_{ub}-C_{cdc,i}\cdot f_{u,cell}\right)+P_{pd}\cdot SA_{cd,i}\cdot \left(C_{cdlum,i}-C_{cdc,i}\cdot f_{u,cell}\right)\right\}/V_{cdc,i}$$

### Appendix A.3. Lumenal Compartments

The urine filtrate was assumed to flow through the kidney lumen. Differential equations for enavogliflozin in the lumenal compartments were as follows:

10. Glomerular Space (i.e., Bowman’s Capsule)

(A10)
$$\frac{d\left(C_{bclum}\right)}{dt}=(GFR\cdot f_{ub}\cdot C_{bcvas}-Q_{ptlum1}\cdot C_{bclum})/V_{bclum}$$

11. Lumen of Proximal Tubule

(A11) $$\frac{d(C_{ptlum,i})}{dt}=\left\{Q_{ptlum,i}\cdot C_{bclum}-Q_{ptlum,i+1}\cdot C_{ptlum,i}-V_{max,reab}\cdot \left(K_{m,reab}+C_{ptlum,i}\right)\cdot C_{ptlum,i}+P_{pd}\;\cdot SA_{pt,i}\cdot \left(C_{ptc,i}\cdot f_{u,cell}-C_{ptlum,i}\right)\right\}/V_{ptlum,i}$$

where i refers to the subsection of the proximal tubule from 1 to 3. For *i* = 2 or 3, $Q_{ptlum,i}$ was multiplied with $C_{ptlum,i-1}$ instead of $C_{bclum}$.

12. Lumen at the Loop of Henle

(A12)
$$\frac{d(C_{lhlum})}{dt}=\left\{Q_{lhlum}\cdot C_{ptlum,3}-Q_{dtlum}\cdot C_{lhlum}+P_{pd}\cdot SA_{lh}\cdot \left(C_{lhc}\cdot f_{u,cell}-C_{lhlum}\right)\right\}/V_{lhlum}$$

13. Lumen at the Distal Tubule

(A13)
$$\frac{d(C_{dtlum})}{dt}=\left\{Q_{dtlum}\cdot C_{lhlum}-Q_{cdlum}\cdot C_{dtlum}+P_{pd}\cdot SA_{dt}\cdot \left(C_{dtc}\cdot f_{u,cell}-C_{dtlum}\right)\right\}/V_{dtlum}$$

14. Lumen at the Collecting Duct

(A14) $$\frac{d(C_{cdlum,i})}{dt}=\left\{Q_{cdlum,i}\cdot C_{dtlum}-Q_{cdlum,i+1}\cdot C_{cdlum,i}+P_{pd}\cdot SA_{cd,i}\cdot \left(C_{cdc,i}\cdot f_{u,cell}-C_{cdlum,i}\right)\right\}/V_{cdlum,i}$$

where i refers to the subsection of the collecting duct from 1 to 2. For *i* = 2, $Q_{cdlum,i}$ was multiplied with $C_{cdlum,i-1}$ instead of $C_{dtlum}$. $Q_{cdlum,3}$ was substituted by $Q_{urine}$.
