# Supplementary material for: Physiologically Based Pharmacokinetic Modelling to Predict Pharmacokinetics of Enavogliflozin, a Sodium-Dependent Glucose Transporter 2 Inhibitor, in Humans
Source: Pharmaceutics. 2023 Mar 14;15(3):942. doi: 10.3390/pharmaceutics15030942 (PMC10058973; doi:10.3390/pharmaceutics15030942)
Supplement: Supplementary file 1 [file pharmaceutics-15-00942-s001.zip › pharmaceutics-2226070-supplementary.pdf]

Table S1. Allometrically scaled effective surface area in humans.

Species	Rat (Body weight: 250 g)[a]		Human (Body weight: 70 kg)	
**Tissue**	**Tissue weight (g)**	**S_{eff} (m^2/g tissue)**	**Tissue weight (kg)**	**S_{eff} (m^2/g tissue)**
**Adipose [b]**	16.6	3.02	15.0	3.60
**Bone [b]**	18.3	2.34	10.0	3.26
**Brain [b]**	1.43	1.67	1.4	1.94
**Gut [b]**	6.75	16.1	1.20	31.9
**Heart [b]**	0.825	18.1	0.329	27.8
**Kidney [b]**	1.83	37.4	0.308	75.3
**Liver**	9.15	14.8	1.80	28.4
**Lung [b]**	1.25	14.4	0.532	21.7
**Muscle [b]**	101	5.71	28.0	9.84
**Skin [b]**	47.6	0.843	2.60	2.41
**Spleen [b]**	0.500	4.56	0.182	7.22

a. The effective surface areas for rats were obtained from the Model 1 of Jeong et al [1].

b. Estimated surface area for the tissues except liver were not used for the PBPK modeling in this study

The exponent between area and weight was obtained from the correlation between body surface (BSA) area and weight (BW) as 0.687 among 10 populations [i.e., $\log(\mathrm{BSA}) = 0.687 \cdot \log(\mathrm{BW}) - 0.989$]. The BSA and BW was obtained from the literature [2].

Table S2. Compartment volumes of the mechanistic kidney model [3].

Compartment	Compartment volume (mL)
Proximal tubule blood 1	2.56
Proximal tubule blood 2	2.56
Proximal tubule blood 3	2.56
Loop of Henle blood	11.78
Distal tubule blood	1.63
Collecting duct blood 1	6.55
Collecting duct blood 2	14.07
Proximal tubule cells 1	18.49
Proximal tubule cells 2	18.49
Proximal tubule cells 3	14.27
Loop of Henle cells	26.8
Distal tubule cells	8.3
Collecting duct cells 1	22.6
Collecting duct cells 2	29.16
Proximal tubule lumen 1	12.38
Proximal tubule lumen 2	12.38
Proximal tubule lumen 3	16.6
Loop of Henle lumen	14.2
Distal tubule lumen	11.35
Collecting duct lumen 1	3.51
Collecting duct lumen 2	18.57
Glomerular blood	7.64
Glomerular space	3.57
Bladder	350

Table S3. Flow rates entering each compartment of the mechanistic kidney model [4, 5].

Compartment	Flow rate (mL/h)
Glomerular filtration rate (GFR)	7200
Proximal tubule lumen	GFR
Loop of Henle lumen	0.36×GFR
Distal Tubule lumen	0.2×GFR
Collecting duct lumen	0.1×GFR
Urine	GFR/120

References

1. Jeong, Y.-S., et al., *Estimation of the minimum permeability coefficient in rats for perfusion-limited tissue distribution in whole-body physiologically-based pharmacokinetics.* European Journal of Pharmaceutics and Biopharmaceutics, 2017. **115**: p. 1-17.
2. Shin, J.-W., I.-C. Seol, and C.-G. Son, *Interpretation of animal dose and human equivalent dose for drug development.* The Journal of Korean Medicine, 2010. **31**(3): p. 1-7.
3. Pletz, J., et al., *A mechanistic model to study the kinetics and toxicity of salicylic acid in the kidney of four virtual individuals.* Computational Toxicology, 2021. **19**: p. 100172.
4. Scotcher, D., et al., *Key to Opening Kidney for In Vitro–In Vivo Extrapolation Entrance in Health and Disease: Part I: In Vitro Systems and Physiological Data.* The AAPS Journal, 2016. **18**(5): p. 1067-1081.
5. Scotcher, D., et al., *Key to Opening Kidney for In Vitro-In Vivo Extrapolation Entrance in Health and Disease: Part II: Mechanistic Models and In Vitro-In Vivo Extrapolation.* The AAPS Journal, 2016. **18**(5): p. 1082-1094.
